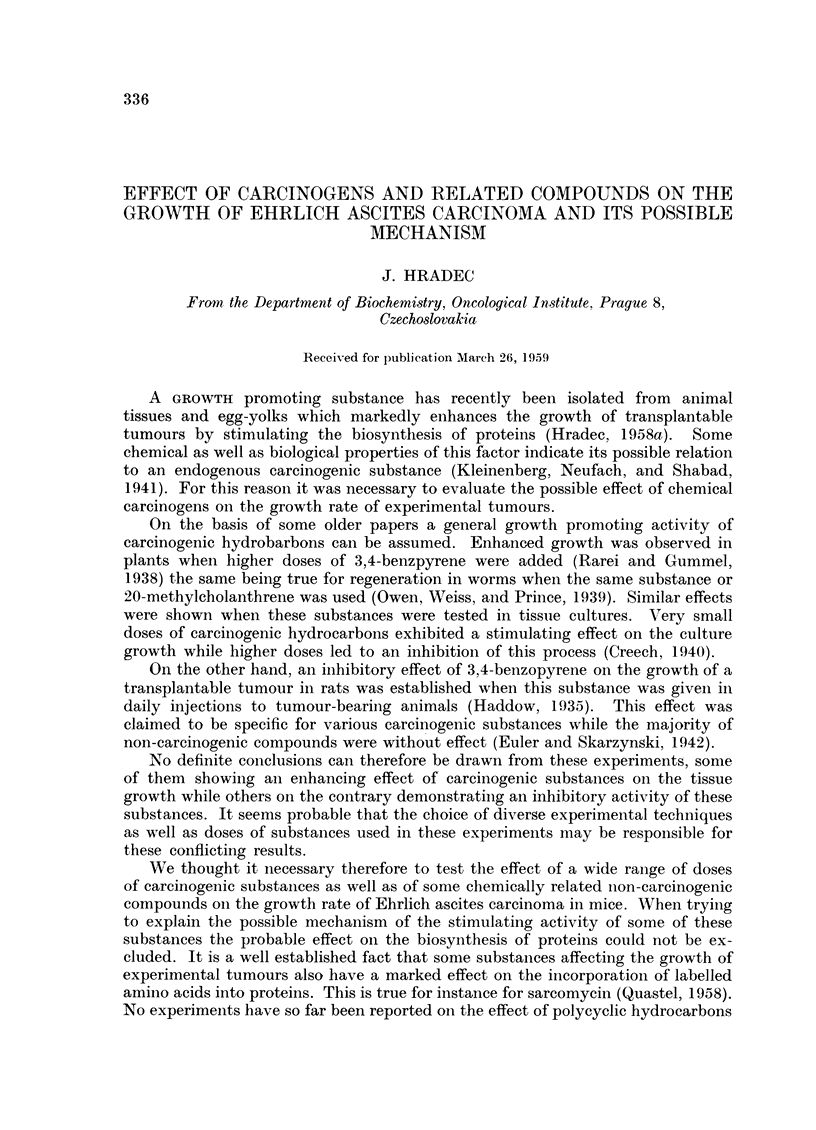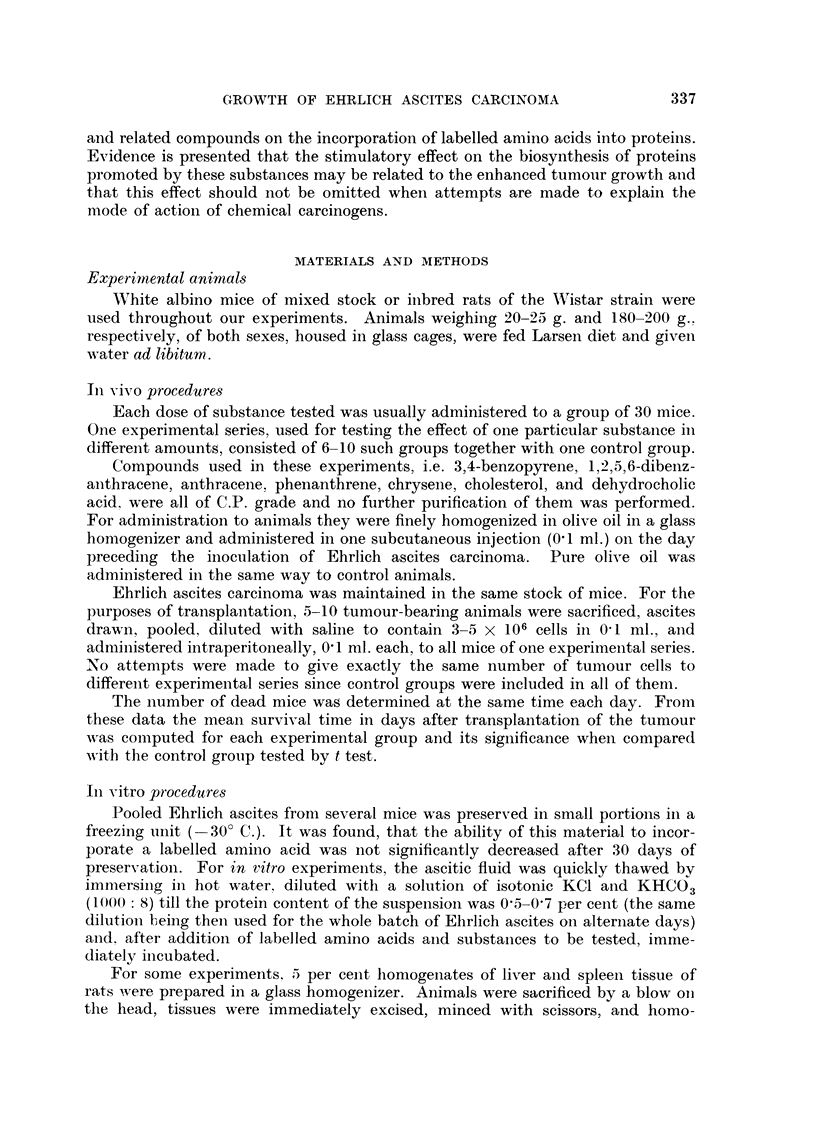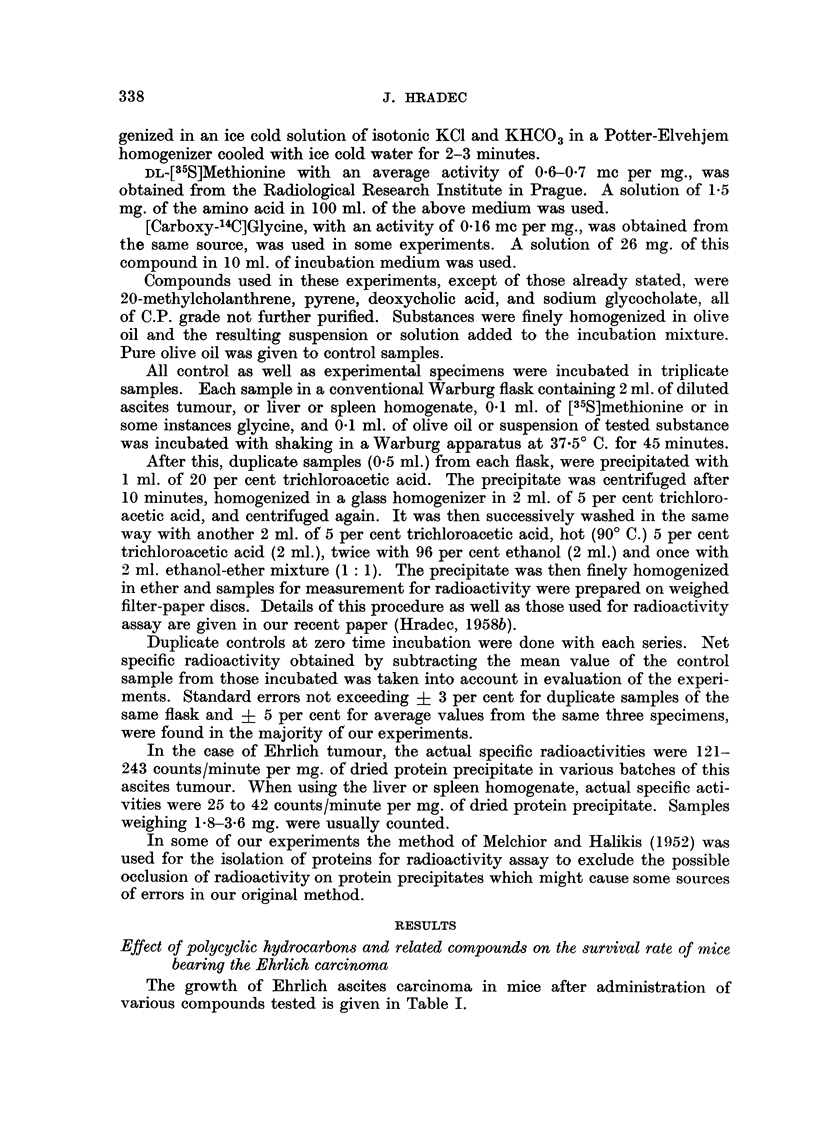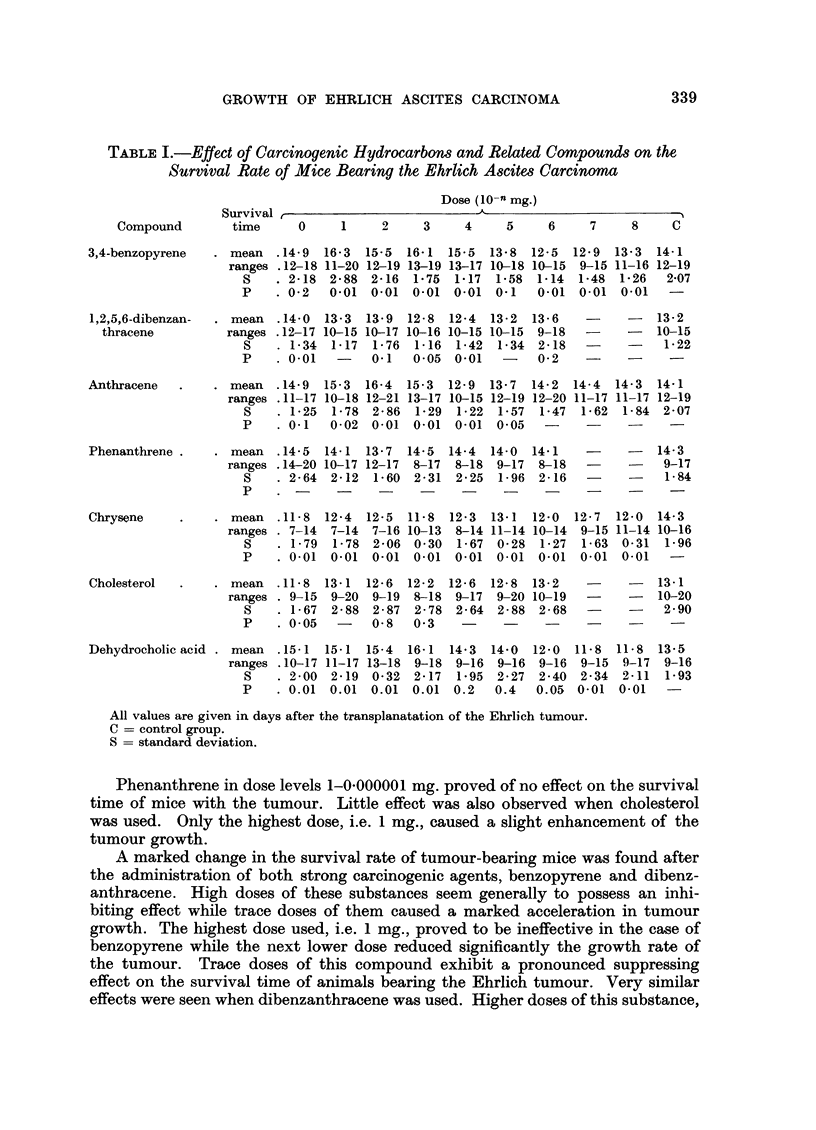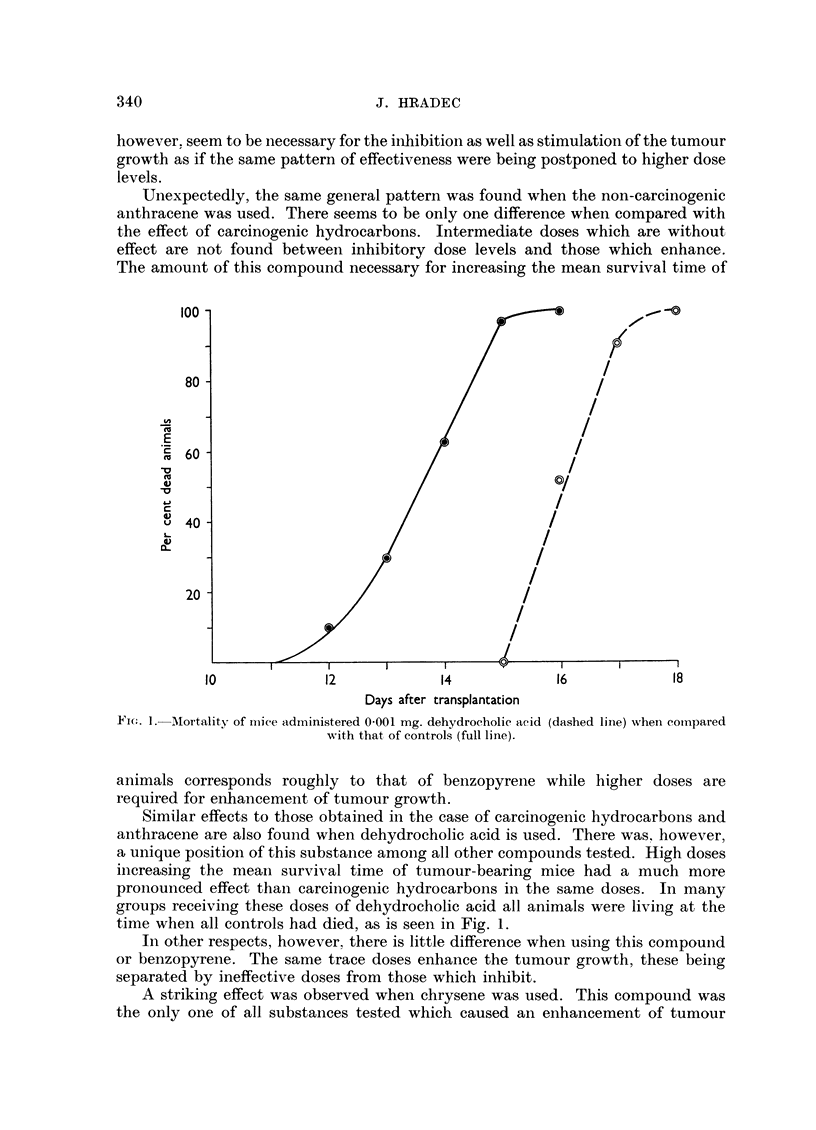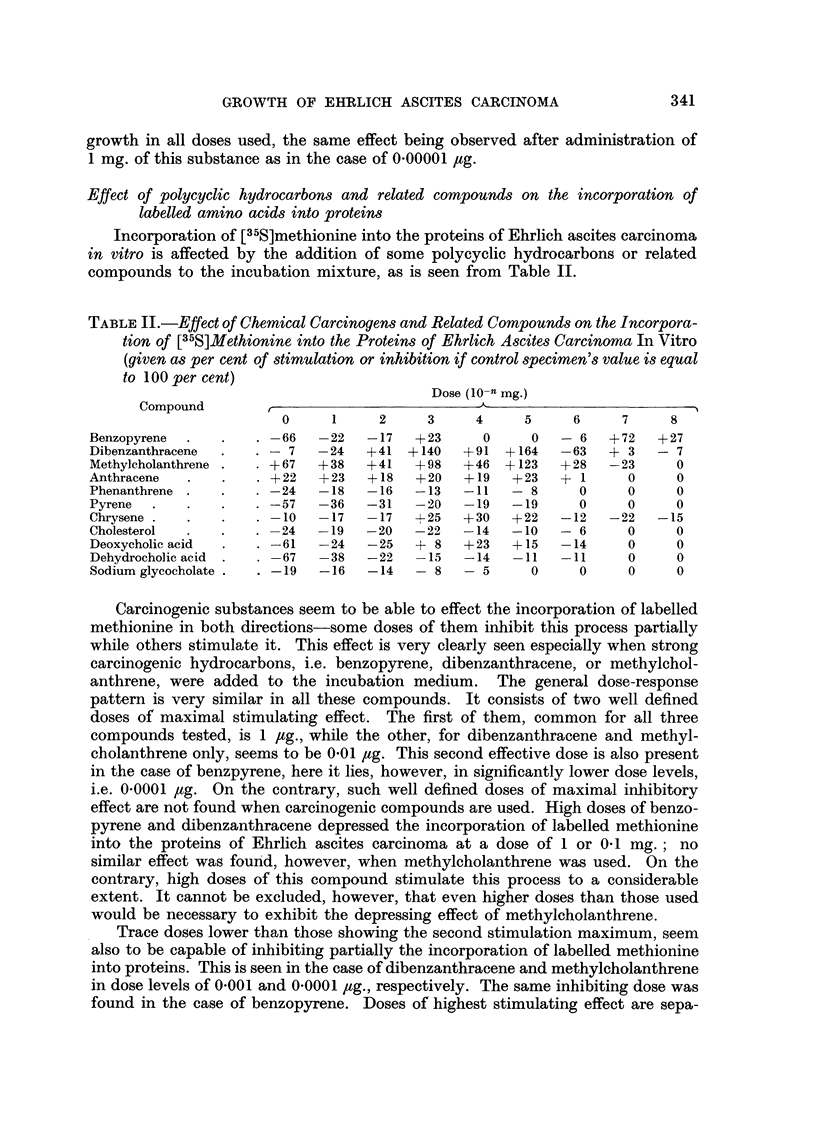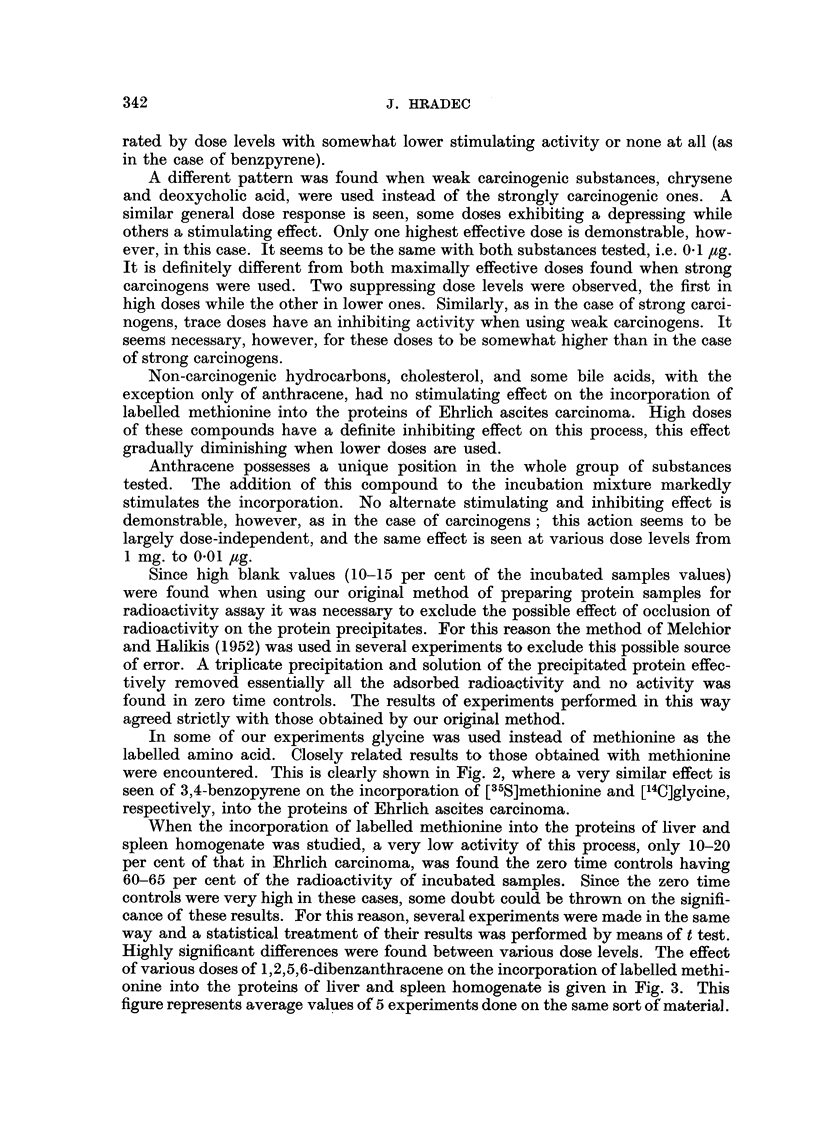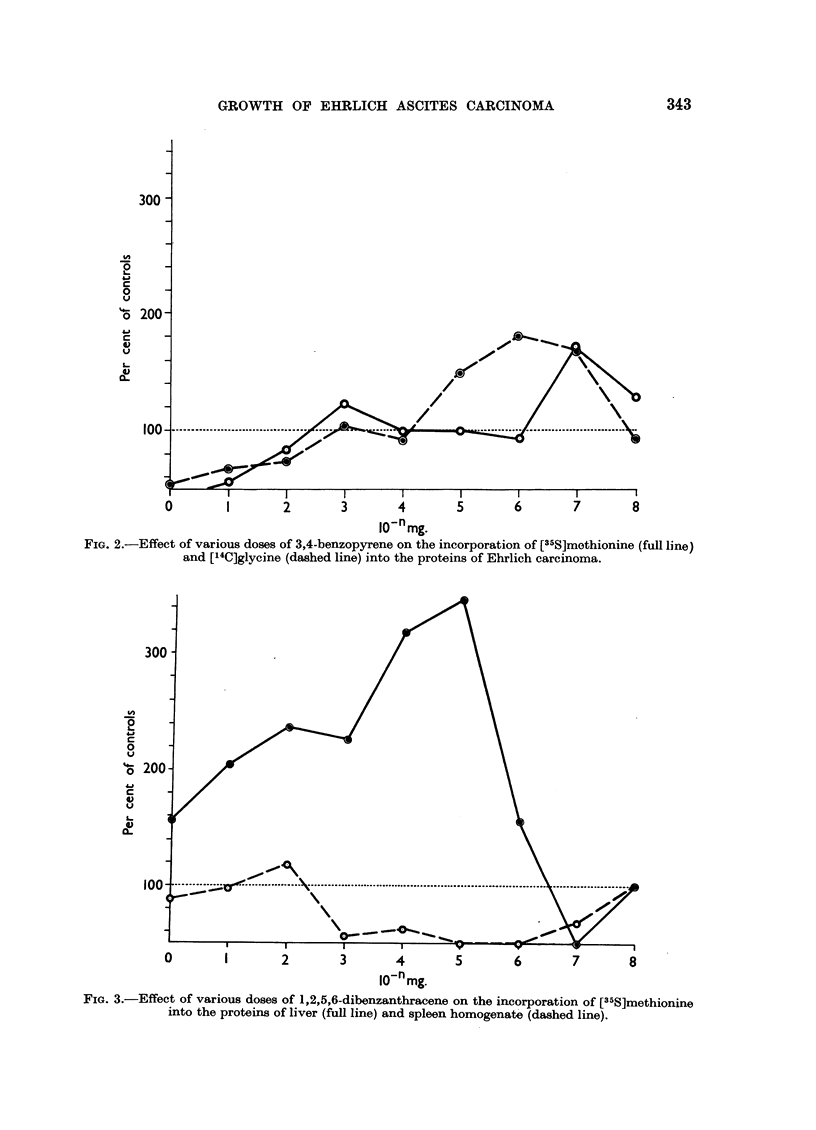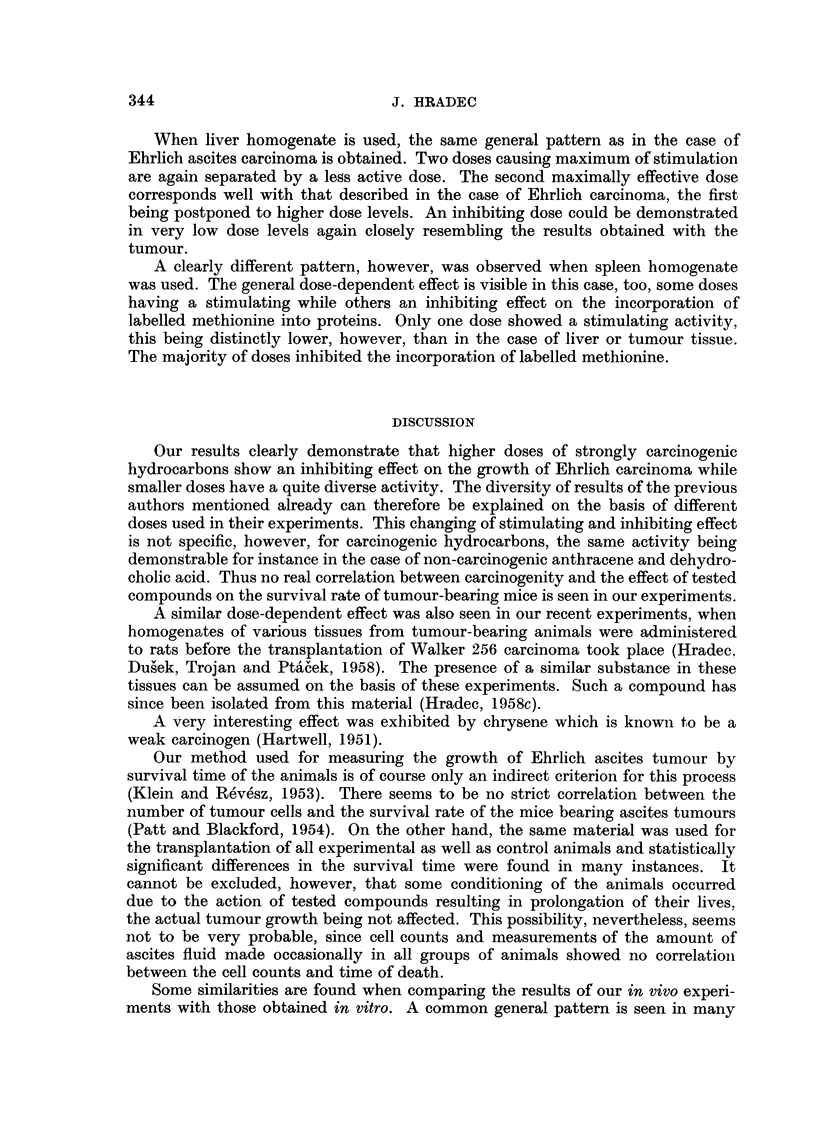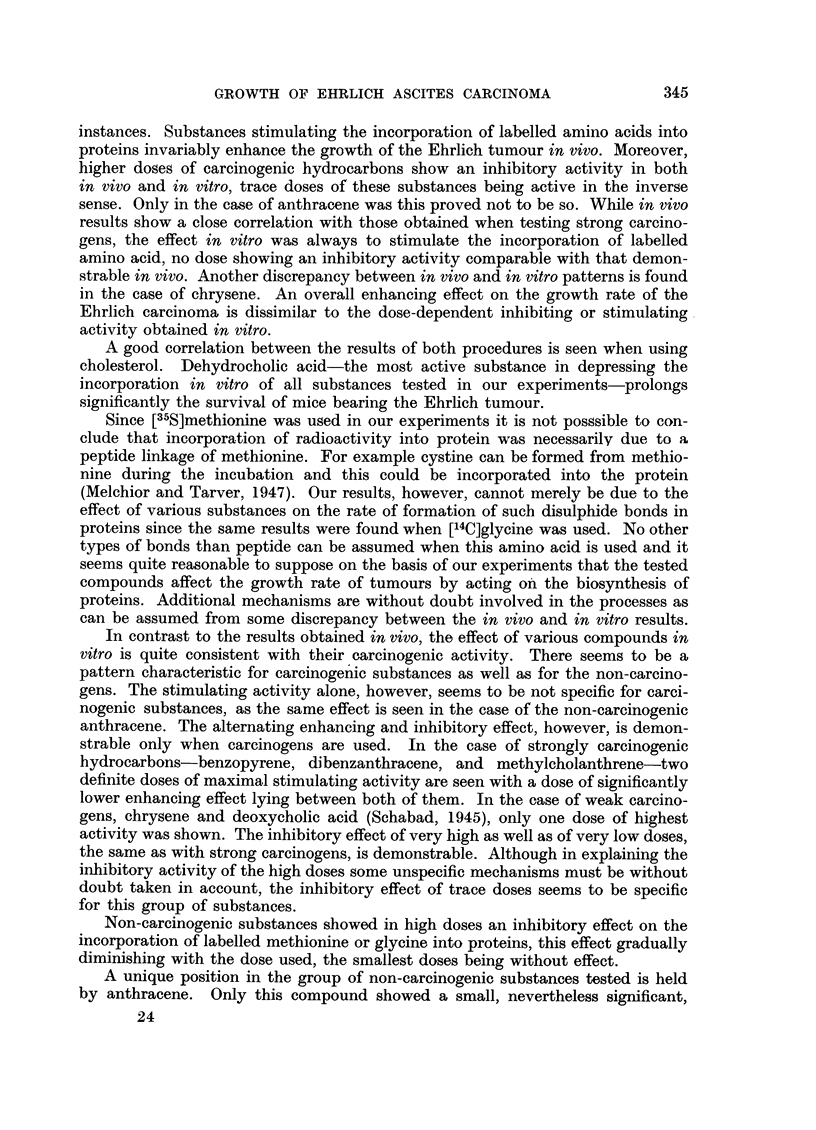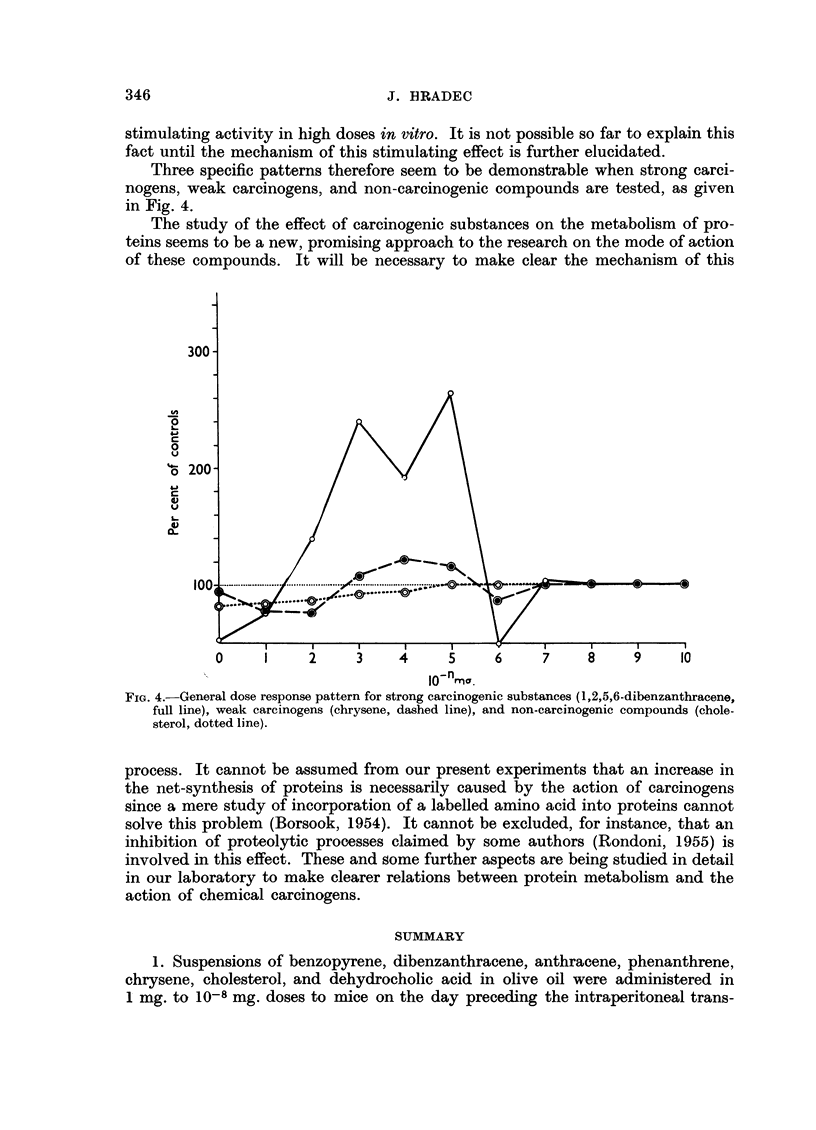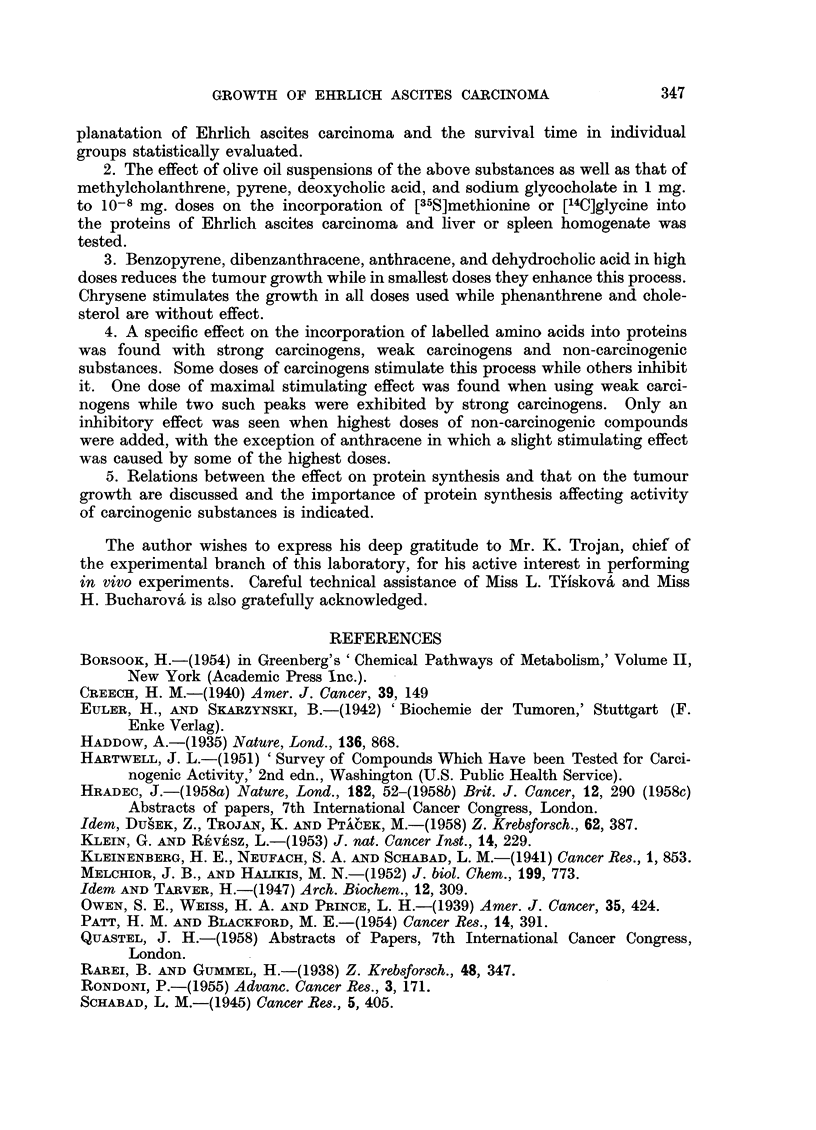# Effect of Carcinogens and Related Compounds on the Growth of Ehrlich Ascites Carcinoma and its Possible Mechanism

**DOI:** 10.1038/bjc.1959.41

**Published:** 1959-06

**Authors:** J. Hradec


### EFFECT OF CARCINOGENS AND RELATED COMPOUNDS ON THE GROWTH OF EHRLICH ASCITES CARCINOMA AND ITS POSSIBLE MECHANISM

J. HRADEC

*From the Department of Biochemistry, Oncological Institute, Prague 8, Czechoslovakia*

Received for publication March 26, 1959

A GROWTH promoting substance has recently been isolated from animal tissues and egg-yolks which markedly enhances the growth of transplantable tumours by stimulating the biosynthesis of proteins (Hradec, 1958*a*). Some chemical as well as biological properties of this factor indicate its possible relation to an endogenous carcinogenic substance (Kleinenberg, Neufach, and Shabad, 1941). For this reason it was necessary to evaluate the possible effect of chemical carcinogens on the growth rate of experimental tumours.

On the basis of some older papers a general growth promoting activity of carcinogenic hydrobarbons can be assumed. Enhanced growth was observed in plants when higher doses of 3,4-benzpyrene were added (Rarei and Gummel, 1938) the same being true for regeneration in worms when the same substance or 20-methylcholanthrene was used (Owen, Weiss, and Prince, 1939). Similar effects were shown when these substances were tested in tissue cultures. Very small doses of carcinogenic hydrocarbons exhibited a stimulating effect on the culture growth while higher doses led to an inhibition of this process (Creech, 1940).

On the other hand, an inhibitory effect of 3,4-benzopyrene on the growth of a transplantable tumour in rats was established when this substance was given in daily injections to tumour-bearing animals (Haddow, 1935). This effect was claimed to be specific for various carcinogenic substances while the majority of non-carcinogenic compounds were without effect (Euler and Skarzynski, 1942).

No definite conclusions can therefore be drawn from these experiments, some of them showing an enhancing effect of carcinogenic substances on the tissue growth while others on the contrary demonstrating an inhibitory activity of these substances. It seems probable that the choice of diverse experimental techniques as well as doses of substances used in these experiments may be responsible for these conflicting results.

We thought it necessary therefore to test the effect of a wide range of doses of carcinogenic substances as well as of some chemically related non-carcinogenic compounds on the growth rate of Ehrlich ascites carcinoma in mice. When trying to explain the possible mechanism of the stimulating activity of some of these substances the probable effect on the biosynthesis of proteins could not be excluded. It is a well established fact that some substances affecting the growth of experimental tumours also have a marked effect on the incorporation of labelled amino acids into proteins. This is true for instance for sarcomycin (Quastel, 1958). No experiments have so far been reported on the effect of polycyclic hydrocarbons and related compounds on the incorporation of labelled amino acids into proteins. Evidence is presented that the stimulatory effect on the biosynthesis of proteins promoted by these substances may be related to the enhanced tumour growth and that this effect should not be omitted when attempts are made to explain the mode of action of chemical carcinogens.

### MATERIALS AND METHODS

### *Experimental animals*

White albino mice of mixed stock or inbred rats of the Wistar strain were used throughout our experiments. Animals weighing 20–25 g. and 180–200 g., respectively, of both sexes, housed in glass cages, were fed Larsen diet and given water *ad libitum*.

### In vivo *procedures*

Each dose of substance tested was usually administered to a group of 30 mice. One experimental series, used for testing the effect of one particular substance in different amounts, consisted of 6–10 such groups together with one control group.

Compounds used in these experiments, i.e. 3,4-benzopyrene, 1,2,5,6-dibenzanthracene, anthracene, phenanthrene, chrysene, cholesterol, and dehydrocholic acid, were all of C.P. grade and no further purification of them was performed. For administration to animals they were finely homogenized in olive oil in a glass homogenizer and administered in one subcutaneous injection (0·1 ml.) on the day preceding the inoculation of Ehrlich ascites carcinoma. Pure olive oil was administered in the same way to control animals.

Ehrlich ascites carcinoma was maintained in the same stock of mice. For the purposes of transplantation, 5–10 tumour-bearing animals were sacrificed, ascites drawn, pooled, diluted with saline to contain $3–5 \times 10^6$ cells in 0·1 ml., and administered intraperitoneally, 0·1 ml. each, to all mice of one experimental series. No attempts were made to give exactly the same number of tumour cells to different experimental series since control groups were included in all of them.

The number of dead mice was determined at the same time each day. From these data the mean survival time in days after transplantation of the tumour was computed for each experimental group and its significance when compared with the control group tested by $t$ test.

### In vitro *procedures*

Pooled Ehrlich ascites from several mice was preserved in small portions in a freezing unit (−30° C.). It was found, that the ability of this material to incorporate a labelled amino acid was not significantly decreased after 30 days of preservation. For *in vitro* experiments, the ascitic fluid was quickly thawed by immersing in hot water, diluted with a solution of isotonic KCl and $KHCO_3$ (1000 : 8) till the protein content of the suspension was 0·5–0·7 per cent (the same dilution being then used for the whole batch of Ehrlich ascites on alternate days) and, after addition of labelled amino acids and substances to be tested, immediately incubated.

For some experiments, 5 per cent homogenates of liver and spleen tissue of rats were prepared in a glass homogenizer. Animals were sacrificed by a blow on the head, tissues were immediately excised, minced with scissors, and homogenized in an ice cold solution of isotonic KCl and $KHCO_3$ in a Potter-Elvehjem homogenizer cooled with ice cold water for 2–3 minutes.

DL-[$^{35}$S]Methionine with an average activity of 0·6–0·7 mc per mg., was obtained from the Radiological Research Institute in Prague. A solution of 1·5 mg. of the amino acid in 100 ml. of the above medium was used.

[Carboxy-$^{14}$C]Glycine, with an activity of 0·16 mc per mg., was obtained from the same source, was used in some experiments. A solution of 26 mg. of this compound in 10 ml. of incubation medium was used.

Compounds used in these experiments, except of those already stated, were 20-methylcholanthrene, pyrene, deoxycholic acid, and sodium glycocholate, all of C.P. grade not further purified. Substances were finely homogenized in olive oil and the resulting suspension or solution added to the incubation mixture. Pure olive oil was given to control samples.

All control as well as experimental specimens were incubated in triplicate samples. Each sample in a conventional Warburg flask containing 2 ml. of diluted ascites tumour, or liver or spleen homogenate, 0·1 ml. of [$^{35}$S]methionine or in some instances glycine, and 0·1 ml. of olive oil or suspension of tested substance was incubated with shaking in a Warburg apparatus at 37·5° C. for 45 minutes.

After this, duplicate samples (0·5 ml.) from each flask, were precipitated with 1 ml. of 20 per cent trichloroacetic acid. The precipitate was centrifuged after 10 minutes, homogenized in a glass homogenizer in 2 ml. of 5 per cent trichloroacetic acid, and centrifuged again. It was then successively washed in the same way with another 2 ml. of 5 per cent trichloroacetic acid, hot (90° C.) 5 per cent trichloroacetic acid (2 ml.), twice with 96 per cent ethanol (2 ml.) and once with 2 ml. ethanol-ether mixture (1 : 1). The precipitate was then finely homogenized in ether and samples for measurement for radioactivity were prepared on weighed filter-paper discs. Details of this procedure as well as those used for radioactivity assay are given in our recent paper (Hradec, 1958*b*).

Duplicate controls at zero time incubation were done with each series. Net specific radioactivity obtained by subtracting the mean value of the control sample from those incubated was taken into account in evaluation of the experiments. Standard errors not exceeding $\pm$ 3 per cent for duplicate samples of the same flask and $\pm$ 5 per cent for average values from the same three specimens, were found in the majority of our experiments.

In the case of Ehrlich tumour, the actual specific radioactivities were 121–243 counts/minute per mg. of dried protein precipitate in various batches of this ascites tumour. When using the liver or spleen homogenate, actual specific activities were 25 to 42 counts/minute per mg. of dried protein precipitate. Samples weighing 1·8–3·6 mg. were usually counted.

In some of our experiments the method of Melchior and Halikis (1952) was used for the isolation of proteins for radioactivity assay to exclude the possible occlusion of radioactivity on protein precipitates which might cause some sources of errors in our original method.

### RESULTS

### *Effect of polycyclic hydrocarbons and related compounds on the survival rate of mice bearing the Ehrlich carcinoma*

The growth of Ehrlich ascites carcinoma in mice after administration of various compounds tested is given in Table I.

TABLE I.—*Effect of Carcinogenic Hydrocarbons and Related Compounds on the Survival Rate of Mice Bearing the Ehrlich Ascites Carcinoma*

| | | Dose ($10^{-n}$ mg.) | | | | | | | | | |
|---|---|---|---|---|---|---|---|---|---|---|---|
| Compound | Survival time | 0 | 1 | 2 | 3 | 4 | 5 | 6 | 7 | 8 | C |
| 3,4-benzopyrene | mean | 14·9 | 16·3 | 15·5 | 16·1 | 15·5 | 13·8 | 12·5 | 12·9 | 13·3 | 14·1 |
| | ranges | 12–18 | 11–20 | 12–19 | 13–19 | 13–17 | 10–18 | 10–15 | 9–15 | 11–16 | 12–19 |
| | S | 2·18 | 2·88 | 2·16 | 1·75 | 1·17 | 1·58 | 1·14 | 1·48 | 1·26 | 2·07 |
| | P | 0·2 | 0·01 | 0·01 | 0·01 | 0·01 | 0·1 | 0·01 | 0·01 | 0·01 | — |
| 1,2,5,6-dibenzanthracene | mean | 14·0 | 13·3 | 13·9 | 12·8 | 12·4 | 13·2 | 13·6 | — | — | 13·2 |
| | ranges | 12–17 | 10–15 | 10–17 | 10–16 | 10–15 | 10–15 | 9–18 | — | — | 10–15 |
| | S | 1·34 | 1·17 | 1·76 | 1·16 | 1·42 | 1·34 | 2·18 | — | — | 1·22 |
| | P | 0·01 | — | 0·1 | 0·05 | 0·01 | — | 0·2 | — | — | — |
| Anthracene | mean | 14·9 | 15·3 | 16·4 | 15·3 | 12·9 | 13·7 | 14·2 | 14·4 | 14·3 | 14·1 |
| | ranges | 11–17 | 10–18 | 12–21 | 13–17 | 10–15 | 12–19 | 12–20 | 11–17 | 11–17 | 12–19 |
| | S | 1·25 | 1·78 | 2·86 | 1·29 | 1·22 | 1·57 | 1·47 | 1·62 | 1·84 | 2·07 |
| | P | 0·1 | 0·02 | 0·01 | 0·01 | 0·01 | 0·05 | — | — | — | — |
| Phenanthrene | mean | 14·5 | 14·1 | 13·7 | 14·5 | 14·4 | 14·0 | 14·1 | — | — | 14·3 |
| | ranges | 14–20 | 10–17 | 12–17 | 8–17 | 8–18 | 9–17 | 8–18 | — | — | 9–17 |
| | S | 2·64 | 2·12 | 1·60 | 2·31 | 2·25 | 1·96 | 2·16 | — | — | 1·84 |
| | P | — | — | — | — | — | — | — | — | — | — |
| Chrysene | mean | 11·8 | 12·4 | 12·5 | 11·8 | 12·3 | 13·1 | 12·0 | 12·7 | 12·0 | 14·3 |
| | ranges | 7–14 | 7–14 | 7–16 | 10–13 | 8–14 | 11–14 | 10–14 | 9–15 | 11–14 | 10–16 |
| | S | 1·79 | 1·78 | 2·06 | 0·30 | 1·67 | 0·28 | 1·27 | 1·63 | 0·31 | 1·96 |
| | P | 0·01 | 0·01 | 0·01 | 0·01 | 0·01 | 0·01 | 0·01 | 0·01 | 0·01 | — |
| Cholesterol | mean | 11·8 | 13·1 | 12·6 | 12·2 | 12·6 | 12·8 | 13·2 | — | — | 13·1 |
| | ranges | 9–15 | 9–20 | 9–19 | 8–18 | 9–17 | 9–20 | 10–19 | — | — | 10–20 |
| | S | 1·67 | 2·88 | 2·87 | 2·78 | 2·64 | 2·88 | 2·68 | — | — | 2·90 |
| | P | 0·05 | — | 0·8 | 0·3 | — | — | — | — | — | — |
| Dehydrocholic acid | mean | 15·1 | 15·1 | 15·4 | 16·1 | 14·3 | 14·0 | 12·0 | 11·8 | 11·8 | 13·5 |
| | ranges | 10–17 | 11–17 | 13–18 | 9–18 | 9–16 | 9–16 | 9–16 | 9–15 | 9–17 | 9–16 |
| | S | 2·00 | 2·19 | 0·32 | 2·17 | 1·95 | 2·27 | 2·40 | 2·34 | 2·11 | 1·93 |
| | P | 0.01 | 0.01 | 0.01 | 0.01 | 0.2 | 0.4 | 0.05 | 0·01 | 0·01 | — |

All values are given in days after the transplanatation of the Ehrlich tumour.
C = control group.
S = standard deviation.

Phenanthrene in dose levels 1–0·000001 mg. proved of no effect on the survival time of mice with the tumour. Little effect was also observed when cholesterol was used. Only the highest dose, i.e. 1 mg., caused a slight enhancement of the tumour growth.

A marked change in the survival rate of tumour-bearing mice was found after the administration of both strong carcinogenic agents, benzopyrene and dibenzanthracene. High doses of these substances seem generally to possess an inhibiting effect while trace doses of them caused a marked acceleration in tumour growth. The highest dose used, i.e. 1 mg., proved to be ineffective in the case of benzopyrene while the next lower dose reduced significantly the growth rate of the tumour. Trace doses of this compound exhibit a pronounced suppressing effect on the survival time of animals bearing the Ehrlich tumour. Very similar effects were seen when dibenzanthracene was used. Higher doses of this substance, however, seem to be necessary for the inhibition as well as stimulation of the tumour growth as if the same pattern of effectiveness were being postponed to higher dose levels.

Unexpectedly, the same general pattern was found when the non-carcinogenic anthracene was used. There seems to be only one difference when compared with the effect of carcinogenic hydrocarbons. Intermediate doses which are without effect are not found between inhibitory dose levels and those which enhance. The amount of this compound necessary for increasing the mean survival time of

FIG. 1.—Mortality of mice administered 0·001 mg. dehydrocholic acid (dashed line) when compared with that of controls (full line).

animals corresponds roughly to that of benzopyrene while higher doses are required for enhancement of tumour growth.

Similar effects to those obtained in the case of carcinogenic hydrocarbons and anthracene are also found when dehydrocholic acid is used. There was, however, a unique position of this substance among all other compounds tested. High doses increasing the mean survival time of tumour-bearing mice had a much more pronounced effect than carcinogenic hydrocarbons in the same doses. In many groups receiving these doses of dehydrocholic acid all animals were living at the time when all controls had died, as is seen in Fig. 1.

In other respects, however, there is little difference when using this compound or benzopyrene. The same trace doses enhance the tumour growth, these being separated by ineffective doses from those which inhibit.

A striking effect was observed when chrysene was used. This compound was the only one of all substances tested which caused an enhancement of tumour growth in all doses used, the same effect being observed after administration of 1 mg. of this substance as in the case of 0·00001 μg.

*Effect of polycyclic hydrocarbons and related compounds on the incorporation of labelled amino acids into proteins*

Incorporation of [$^{35}$S]methionine into the proteins of Ehrlich ascites carcinoma *in vitro* is affected by the addition of some polycyclic hydrocarbons or related compounds to the incubation mixture, as is seen from Table II.

TABLE II.—*Effect of Chemical Carcinogens and Related Compounds on the Incorporation of* [$^{35}$S]*Methionine into the Proteins of Ehrlich Ascites Carcinoma* In Vitro *(given as per cent of stimulation or inhibition if control specimen's value is equal to* 100 *per cent)*

| Compound | Dose ($10^{-n}$ mg.) | | | | | | | | |
|---|---|---|---|---|---|---|---|---|---|
| | 0 | 1 | 2 | 3 | 4 | 5 | 6 | 7 | 8 |
| Benzopyrene | −66 | −22 | −17 | +23 | 0 | 0 | − 6 | +72 | +27 |
| Dibenzanthracene | − 7 | −24 | +41 | +140 | +91 | +164 | −63 | + 3 | − 7 |
| Methylcholanthrene | +67 | +38 | +41 | +98 | +46 | +123 | +28 | −23 | 0 |
| Anthracene | +22 | +23 | +18 | +20 | +19 | +23 | + 1 | 0 | 0 |
| Phenanthrene | −24 | −18 | −16 | −13 | −11 | − 8 | 0 | 0 | 0 |
| Pyrene | −57 | −36 | −31 | −20 | −19 | −19 | 0 | 0 | 0 |
| Chrysene | −10 | −17 | −17 | +25 | +30 | +22 | −12 | −22 | −15 |
| Cholesterol | −24 | −19 | −20 | −22 | −14 | −10 | − 6 | 0 | 0 |
| Deoxycholic acid | −61 | −24 | −25 | + 8 | +23 | +15 | −14 | 0 | 0 |
| Dehydrocholic acid | −67 | −38 | −22 | −15 | −14 | −11 | −11 | 0 | 0 |
| Sodium glycocholate | −19 | −16 | −14 | − 8 | − 5 | 0 | 0 | 0 | 0 |

Carcinogenic substances seem to be able to effect the incorporation of labelled methionine in both directions—some doses of them inhibit this process partially while others stimulate it. This effect is very clearly seen especially when strong carcinogenic hydrocarbons, i.e. benzopyrene, dibenzanthracene, or methylcholanthrene, were added to the incubation medium. The general dose-response pattern is very similar in all these compounds. It consists of two well defined doses of maximal stimulating effect. The first of them, common for all three compounds tested, is 1 μg., while the other, for dibenzanthracene and methylcholanthrene only, seems to be 0·01 μg. This second effective dose is also present in the case of benzpyrene, here it lies, however, in significantly lower dose levels, i.e. 0·0001 μg. On the contrary, such well defined doses of maximal inhibitory effect are not found when carcinogenic compounds are used. High doses of benzopyrene and dibenzanthracene depressed the incorporation of labelled methionine into the proteins of Ehrlich ascites carcinoma at a dose of 1 or 0·1 mg. ; no similar effect was found, however, when methylcholanthrene was used. On the contrary, high doses of this compound stimulate this process to a considerable extent. It cannot be excluded, however, that even higher doses than those used would be necessary to exhibit the depressing effect of methylcholanthrene.

Trace doses lower than those showing the second stimulation maximum, seem also to be capable of inhibiting partially the incorporation of labelled methionine into proteins. This is seen in the case of dibenzanthracene and methylcholanthrene in dose levels of 0·001 and 0·0001 μg., respectively. The same inhibiting dose was found in the case of benzopyrene. Doses of highest stimulating effect are separated by dose levels with somewhat lower stimulating activity or none at all (as in the case of benzpyrene).

A different pattern was found when weak carcinogenic substances, chrysene and deoxycholic acid, were used instead of the strongly carcinogenic ones. A similar general dose response is seen, some doses exhibiting a depressing while others a stimulating effect. Only one highest effective dose is demonstrable, however, in this case. It seems to be the same with both substances tested, i.e. 0·1 μg. It is definitely different from both maximally effective doses found when strong carcinogens were used. Two suppressing dose levels were observed, the first in high doses while the other in lower ones. Similarly, as in the case of strong carcinogens, trace doses have an inhibiting activity when using weak carcinogens. It seems necessary, however, for these doses to be somewhat higher than in the case of strong carcinogens.

Non-carcinogenic hydrocarbons, cholesterol, and some bile acids, with the exception only of anthracene, had no stimulating effect on the incorporation of labelled methionine into the proteins of Ehrlich ascites carcinoma. High doses of these compounds have a definite inhibiting effect on this process, this effect gradually diminishing when lower doses are used.

Anthracene possesses a unique position in the whole group of substances tested. The addition of this compound to the incubation mixture markedly stimulates the incorporation. No alternate stimulating and inhibiting effect is demonstrable, however, as in the case of carcinogens ; this action seems to be largely dose-independent, and the same effect is seen at various dose levels from 1 mg. to 0·01 μg.

Since high blank values (10–15 per cent of the incubated samples values) were found when using our original method of preparing protein samples for radioactivity assay it was necessary to exclude the possible effect of occlusion of radioactivity on the protein precipitates. For this reason the method of Melchior and Halikis (1952) was used in several experiments to exclude this possible source of error. A triplicate precipitation and solution of the precipitated protein effectively removed essentially all the adsorbed radioactivity and no activity was found in zero time controls. The results of experiments performed in this way agreed strictly with those obtained by our original method.

In some of our experiments glycine was used instead of methionine as the labelled amino acid. Closely related results to those obtained with methionine were encountered. This is clearly shown in Fig. 2, where a very similar effect is seen of 3,4-benzopyrene on the incorporation of [$^{35}$S]methionine and [$^{14}$C]glycine, respectively, into the proteins of Ehrlich ascites carcinoma.

When the incorporation of labelled methionine into the proteins of liver and spleen homogenate was studied, a very low activity of this process, only 10–20 per cent of that in Ehrlich carcinoma, was found the zero time controls having 60–65 per cent of the radioactivity of incubated samples. Since the zero time controls were very high in these cases, some doubt could be thrown on the significance of these results. For this reason, several experiments were made in the same way and a statistical treatment of their results was performed by means of *t* test. Highly significant differences were found between various dose levels. The effect of various doses of 1,2,5,6-dibenzanthracene on the incorporation of labelled methionine into the proteins of liver and spleen homogenate is given in Fig. 3. This figure represents average values of 5 experiments done on the same sort of material.

FIG. 2.—Effect of various doses of 3,4-benzopyrene on the incorporation of [$^{35}$S]methionine (full line) and [$^{14}$C]glycine (dashed line) into the proteins of Ehrlich carcinoma.

FIG. 3.—Effect of various doses of 1,2,5,6-dibenzanthracene on the incorporation of [$^{35}$S]methionine into the proteins of liver (full line) and spleen homogenate (dashed line).

When liver homogenate is used, the same general pattern as in the case of Ehrlich ascites carcinoma is obtained. Two doses causing maximum of stimulation are again separated by a less active dose. The second maximally effective dose corresponds well with that described in the case of Ehrlich carcinoma, the first being postponed to higher dose levels. An inhibiting dose could be demonstrated in very low dose levels again closely resembling the results obtained with the tumour.

A clearly different pattern, however, was observed when spleen homogenate was used. The general dose-dependent effect is visible in this case, too, some doses having a stimulating while others an inhibiting effect on the incorporation of labelled methionine into proteins. Only one dose showed a stimulating activity, this being distinctly lower, however, than in the case of liver or tumour tissue. The majority of doses inhibited the incorporation of labelled methionine.

### DISCUSSION

Our results clearly demonstrate that higher doses of strongly carcinogenic hydrocarbons show an inhibiting effect on the growth of Ehrlich carcinoma while smaller doses have a quite diverse activity. The diversity of results of the previous authors mentioned already can therefore be explained on the basis of different doses used in their experiments. This changing of stimulating and inhibiting effect is not specific, however, for carcinogenic hydrocarbons, the same activity being demonstrable for instance in the case of non-carcinogenic anthracene and dehydrocholic acid. Thus no real correlation between carcinogenity and the effect of tested compounds on the survival rate of tumour-bearing mice is seen in our experiments.

A similar dose-dependent effect was also seen in our recent experiments, when homogenates of various tissues from tumour-bearing animals were administered to rats before the transplantation of Walker 256 carcinoma took place (Hradec, Dušek, Trojan and Ptáček, 1958). The presence of a similar substance in these tissues can be assumed on the basis of these experiments. Such a compound has since been isolated from this material (Hradec, 1958*c*).

A very interesting effect was exhibited by chrysene which is known to be a weak carcinogen (Hartwell, 1951).

Our method used for measuring the growth of Ehrlich ascites tumour by survival time of the animals is of course only an indirect criterion for this process (Klein and Révész, 1953). There seems to be no strict correlation between the number of tumour cells and the survival rate of the mice bearing ascites tumours (Patt and Blackford, 1954). On the other hand, the same material was used for the transplantation of all experimental as well as control animals and statistically significant differences in the survival time were found in many instances. It cannot be excluded, however, that some conditioning of the animals occurred due to the action of tested compounds resulting in prolongation of their lives, the actual tumour growth being not affected. This possibility, nevertheless, seems not to be very probable, since cell counts and measurements of the amount of ascites fluid made occasionally in all groups of animals showed no correlation between the cell counts and time of death.

Some similarities are found when comparing the results of our *in vivo* experiments with those obtained *in vitro*. A common general pattern is seen in many instances. Substances stimulating the incorporation of labelled amino acids into proteins invariably enhance the growth of the Ehrlich tumour *in vivo*. Moreover, higher doses of carcinogenic hydrocarbons show an inhibitory activity in both *in vivo* and *in vitro*, trace doses of these substances being active in the inverse sense. Only in the case of anthracene was this proved not to be so. While *in vivo* results show a close correlation with those obtained when testing strong carcinogens, the effect *in vitro* was always to stimulate the incorporation of labelled amino acid, no dose showing an inhibitory activity comparable with that demonstrable *in vivo*. Another discrepancy between *in vivo* and *in vitro* patterns is found in the case of chrysene. An overall enhancing effect on the growth rate of the Ehrlich carcinoma is dissimilar to the dose-dependent inhibiting or stimulating activity obtained *in vitro*.

A good correlation between the results of both procedures is seen when using cholesterol. Dehydrocholic acid—the most active substance in depressing the incorporation *in vitro* of all substances tested in our experiments—prolongs significantly the survival of mice bearing the Ehrlich tumour.

Since [$^{35}$S]methionine was used in our experiments it is not posssible to conclude that incorporation of radioactivity into protein was necessarily due to a peptide linkage of methionine. For example cystine can be formed from methionine during the incubation and this could be incorporated into the protein (Melchior and Tarver, 1947). Our results, however, cannot merely be due to the effect of various substances on the rate of formation of such disulphide bonds in proteins since the same results were found when [$^{14}$C]glycine was used. No other types of bonds than peptide can be assumed when this amino acid is used and it seems quite reasonable to suppose on the basis of our experiments that the tested compounds affect the growth rate of tumours by acting on the biosynthesis of proteins. Additional mechanisms are without doubt involved in the processes as can be assumed from some discrepancy between the *in vivo* and *in vitro* results.

In contrast to the results obtained *in vivo*, the effect of various compounds *in vitro* is quite consistent with their carcinogenic activity. There seems to be a pattern characteristic for carcinogenic substances as well as for the non-carcinogens. The stimulating activity alone, however, seems to be not specific for carcinogenic substances, as the same effect is seen in the case of the non-carcinogenic anthracene. The alternating enhancing and inhibitory effect, however, is demonstrable only when carcinogens are used. In the case of strongly carcinogenic hydrocarbons—benzopyrene, dibenzanthracene, and methylcholanthrene—two definite doses of maximal stimulating activity are seen with a dose of significantly lower enhancing effect lying between both of them. In the case of weak carcinogens, chrysene and deoxycholic acid (Schabad, 1945), only one dose of highest activity was shown. The inhibitory effect of very high as well as of very low doses, the same as with strong carcinogens, is demonstrable. Although in explaining the inhibitory activity of the high doses some unspecific mechanisms must be without doubt taken in account, the inhibitory effect of trace doses seems to be specific for this group of substances.

Non-carcinogenic substances showed in high doses an inhibitory effect on the incorporation of labelled methionine or glycine into proteins, this effect gradually diminishing with the dose used, the smallest doses being without effect.

A unique position in the group of non-carcinogenic substances tested is held by anthracene. Only this compound showed a small, nevertheless significant, stimulating activity in high doses *in vitro*. It is not possible so far to explain this fact until the mechanism of this stimulating effect is further elucidated.

Three specific patterns therefore seem to be demonstrable when strong carcinogens, weak carcinogens, and non-carcinogenic compounds are tested, as given in Fig. 4.

The study of the effect of carcinogenic substances on the metabolism of proteins seems to be a new, promising approach to the research on the mode of action of these compounds. It will be necessary to make clear the mechanism of this

FIG. 4.—General dose response pattern for strong carcinogenic substances (1,2,5,6-dibenzanthracene, full line), weak carcinogens (chrysene, dashed line), and non-carcinogenic compounds (cholesterol, dotted line).

process. It cannot be assumed from our present experiments that an increase in the net-synthesis of proteins is necessarily caused by the action of carcinogens since a mere study of incorporation of a labelled amino acid into proteins cannot solve this problem (Borsook, 1954). It cannot be excluded, for instance, that an inhibition of proteolytic processes claimed by some authors (Rondoni, 1955) is involved in this effect. These and some further aspects are being studied in detail in our laboratory to make clearer relations between protein metabolism and the action of chemical carcinogens.

### SUMMARY

1. Suspensions of benzopyrene, dibenzanthracene, anthracene, phenanthrene, chrysene, cholesterol, and dehydrocholic acid in olive oil were administered in 1 mg. to $10^{-8}$ mg. doses to mice on the day preceding the intraperitoneal transplanatation of Ehrlich ascites carcinoma and the survival time in individual groups statistically evaluated.

2. The effect of olive oil suspensions of the above substances as well as that of methylcholanthrene, pyrene, deoxycholic acid, and sodium glycocholate in 1 mg. to $10^{-8}$ mg. doses on the incorporation of [$^{35}$S]methionine or [$^{14}$C]glycine into the proteins of Ehrlich ascites carcinoma and liver or spleen homogenate was tested.

3. Benzopyrene, dibenzanthracene, anthracene, and dehydrocholic acid in high doses reduces the tumour growth while in smallest doses they enhance this process. Chrysene stimulates the growth in all doses used while phenanthrene and cholesterol are without effect.

4. A specific effect on the incorporation of labelled amino acids into proteins was found with strong carcinogens, weak carcinogens and non-carcinogenic substances. Some doses of carcinogens stimulate this process while others inhibit it. One dose of maximal stimulating effect was found when using weak carcinogens while two such peaks were exhibited by strong carcinogens. Only an inhibitory effect was seen when highest doses of non-carcinogenic compounds were added, with the exception of anthracene in which a slight stimulating effect was caused by some of the highest doses.

5. Relations between the effect on protein synthesis and that on the tumour growth are discussed and the importance of protein synthesis affecting activity of carcinogenic substances is indicated.

The author wishes to express his deep gratitude to Mr. K. Trojan, chief of the experimental branch of this laboratory, for his active interest in performing *in vivo* experiments. Careful technical assistance of Miss L. Třísková and Miss H. Bucharová is also gratefully acknowledged.

### REFERENCES

Borsook, H.—(1954) in Greenberg's 'Chemical Pathways of Metabolism,' Volume II, New York (Academic Press Inc.).
Creech, H. M.—(1940) *Amer. J. Cancer*, **39**, 149
Euler, H., and Skarzynski, B.—(1942) 'Biochemie der Tumoren,' Stuttgart (F. Enke Verlag).
Haddow, A.—(1935) *Nature, Lond.*, **136**, 868.
Hartwell, J. L.—(1951) 'Survey of Compounds Which Have been Tested for Carcinogenic Activity,' 2nd edn., Washington (U.S. Public Health Service).
Hradec, J.—(1958*a*) *Nature, Lond.*, **182**, 52–(1958*b*) *Brit. J. Cancer*, **12**, 290 (1958*c*) Abstracts of papers, 7th International Cancer Congress, London.
*Idem*, Dušek, Z., Trojan, K. and Ptáček, M.—(1958) *Z. Krebsforsch.*, **62**, 387.
Klein, G. and Révész, L.—(1953) *J. nat. Cancer Inst.*, **14**, 229.
Kleinenberg, H. E., Neufach, S. A. and Schabad, L. M.—(1941) *Cancer Res.*, **1**, 853.
Melchior, J. B., and Halikis, M. N.—(1952) *J. biol. Chem.*, **199**, 773.
*Idem* and Tarver, H.—(1947) *Arch. Biochem.*, **12**, 309.
Owen, S. E., Weiss, H. A. and Prince, L. H.—(1939) *Amer. J. Cancer*, **35**, 424.
Patt, H. M. and Blackford, M. E.—(1954) *Cancer Res.*, **14**, 391.
Quastel, J. H.—(1958) Abstracts of Papers, 7th International Cancer Congress, London.
Rarei, B. and Gummel, H.—(1938) *Z. Krebsforsch.*, **48**, 347.
Rondoni, P.—(1955) *Advanc. Cancer Res.*, **3**, 171.
Schabad, L. M.—(1945) *Cancer Res.*, **5**, 405.